# Evaluation of the knowledge, attitudes and practices of students at the University of Abomey-Calavi on rabies in Benin Republic, West Africa

**DOI:** 10.11604/pamj.2021.38.235.27485

**Published:** 2021-03-04

**Authors:** Philippe Sessou, Nestor Noudeke, Deborah Janine Thomson, David Salako, Souaïbou Farougou

**Affiliations:** 1 Research Unit on Communicable Diseases, Polytechnic School of Abomey-Calavi, University of Abomey-Calavi, Cotonou, Benin,; 2 One Health Institute, University of California, California, USA

**Keywords:** Knowledge, attitudes, rabies, Benin, education, zoonotic disease, one health, one health education

## Abstract

**Introduction:**

rabies is a vaccine-preventable viral zoonotic disease. Once clinical symptoms appear, rabies is fatal in almost 100% of cases. The objective of this study was to evaluate the knowledge, attitudes and practices of students at the University of Abomey-Calavi in Benin on rabies in order to explore the factors that promote the occurrence of this zoonosis.

**Methods:**

for this purpose, a descriptive cross-sectional survey was conducted among 263 randomly-selected students. The collected data were analyzed by R software with logistic regression.

**Results:**

out of all the 263 respondents, 53.2% (n=140) of the students claimed to have heard of canine rabies, compared to 47.5% (n=125) for human rabies. Stray dogs were recognized by 49.0% (n=129) as a prevailing source of rabies infection in people; bites from these dogs were considered as a means of rabies contagion (41.4%; n=109) and vaccination of dogs was considered by 32.7% (n=86) as a means of rabies control in both people and dogs. In case of a dog bite, 60.5% (n=159) of respondents would visit a western medicine human clinic first. For the fate of the biting dog, 18.6% (n=49) and 27.4% (n=72) of respondents, respectively, prefer to euthanize the dog or take the dog to the veterinarian for observation. Regarding the perceived consequences of inaction after a dog bite, 58.2% (n=140) mentioned the risk of rabies. Students in human or animal health were 3 times more aware on rabies.

**Conclusion:**

this study identifies the gaps in students´ knowledge, attitudes and practices about effective rabies prevention and control. It will therefore be necessary to intensify awareness and education campaigns among students who could be a good relay of information to other members in their communities.

## Introduction

Rabies is a neglected tropical disease affecting mainly poor and vulnerable populations living in isolated rural areas. It is clinically characterized by the appearance of a pattern of encephalitis which is almost always fatal after a patient experiences great suffering [1,2]. This animal-human communicable disease represents a significant global health and public health burden [3,4]. Indeed, treatment of a bitten person can cost 40-50 USD and represents a catastrophic financial burden for affected families, whose average daily income is around 1-2 USD per person [2]. According to the World Health Organization (WHO), more than 60,000 human deaths are caused by rabies each year and children (aged 5-14 years) are the most frequent victims [2,5]. Ninety five percent (95%) of global human rabies deaths occur in Africa and Asia and 99% of rabies cases are associated with dog bites. This disease burden is disproportionally found in rural and impoverished communities, with approximately half of cases attributable to children under 15 [2].

In Benin, the fight against rabies still faces enormous obstacles. Many human rabies deaths are associated with local animal outbreaks [6]. A retrospective study conducted in 2019 by the primary author and collaborators in southern Benin [5] revealed that 770 out of 900 biting dogs counted between 2012-2017 in the cities of Cotonou, Abomey-Calavi and Ouidah were put under observation after the biting incident. During this observation period, 6.88% of canine cases were suspected of canine rabies. The results of the analysis of curative care registers in the surveyed human medical centers revealed that 1436 cases of dog bites were recorded and 287 clinical cases of human rabies (20% of total dog bite cases) were fatal. Given the high number of deaths due to this zoonosis and its socio-economic importance, the implementation of an effective strategic plan for its control is necessary for its eradication [5].

Prevention of rabies requires better knowledge of the disease and appropriate attitudes and practices that should be adopted in case of a dog bite among the general public and especially students considered as “health relay”, a precious aid to prevention of zoonoses. To our knowledge, no formal KAP study has been published to date in Benin [7]. This current study aimed to evaluate the knowledge, attitudes and practices of students at the University of Abomey-Calavi in relation to rabies.

## Methods

**Study design and setting:** data collection for this study was conducted at the student level at the University of Abomey-Calavi (UAC) from August to September, 2020. This university, located in Southern Benin, is a government-funded institute under the supervision of the Ministry of Higher Education and Scientific Research (MESRS). It encompasses thirty-three (33) training entities including five (5) faculties of classical training, eighteen (18) schools and vocational training institutes and ten (10) doctoral schools. In 2016, this university had 68,978 students [8]. A cross-sectional descriptive study was conducted among the student community to study the knowledge, attitudes and practices (KAP) of learners regarding rabies.

**Study population:** this study was conducted on the student community of the University of Abomey-Calavi campuses including the Abomey-Calavi campus and the Faculty of Health Sciences in Cotonou. Students were randomly selected from all faculties located on the indexed campuses. A total of 263 students of various majors and disciplines (including medicine, veterinary medicine, social sciences, economics, management, law, and business) were surveyed.

**Data collection:** information assessing students´ knowledge, attitudes and practices regarding rabies prevention were collected through a well-structured 50-questions survey. The questionnaire was tested and validated prior to be used in this study. Participants, after having freely given their consent and being told their answers are untraceable, were asked to volunteer and give correct answers to each question in a 2-part questionnaire. The first section focused on the volunteer respondent´s socio-demographic information (i.e. gender, age, household size, education and occupation). The second section evaluated rabies-specific knowledge, attitudes and practices. Questions addressed the cause of rabies, mode of transmission, clinical signs in people and animals, as well as the respondent´s practices and attitudes to control and prevent rabies. Data were collected through in-person interviews.

**Statistical analysis:** after collection, the data were entered into a spreadsheet made with Microsoft Access. Then, it was exported to Microsoft Excel to be evaluated for completeness. Incomplete and inconsistent data were corrected where possible. Two new variables (each with 2 modalities of either “sufficient” or “insufficient”) were created in the database taking into account: (1) all the variables regarding rabies knowledge and (2) all the variables regarding attitudes and practices about rabies prevention. To do this evaluation, all the modalities proposed in the questionnaire for each of the variables (Knowledge, Attitudes and Practices) were given a score of 1 if they were properly answered and a score of 0 if they were incorrect. Then, for each variable (Knowledge, Attitudes and Practices), the incorrect modalities proposed in the questionnaire were excluded while the scores (0 or 1) of the correct modalities proposed for each variable (Knowledge, Attitudes and Practices) were added. Therefore, for the variable “Knowledge”, the total scores vary from 0 to 7 with an average of 4, so that respondents with a score strictly below 4 are considered to have poor (“insufficient”) knowledge, while those with a score of 4 or more are considered to have good (“sufficient”) knowledge. For the “Attitudes and Practices” variables, overall scores ranged from 0 to 4 with an average of 3. Respondents scoring strictly below 3 were considered to have poor (“insufficient”) attitudes and practices, while those scoring 3 or more were considered to have good (“sufficient”) attitudes and practices. Finally, after checking and cleaning, the data were coded and then exported to the statistical software R version 3.3.3 (Package Epicalc). The frequency distributions of the dependent and independent variables were carried out using descriptive statistical techniques (frequency and percentage). Univariable and multivariable logistic regression analyses were performed exploring factors associated with levels of knowledge (adequate vs inadequate), with attitudes and practices. Variables with a p-value less than 0.2 in the univariable analysis were included in the model of the multivariable analysis.

**Ethical considerations:** ethical approval was obtained from Ethical Review Board of the Department of Animal Heath and Production, University of Abomey-Calavi. Furthermore, informed consent was obtained from the respondents. Anonymity and Informed consent were required from each participant who was aware that participation is voluntary and there was no consequence for non-participation. All informations obtained were kept confidential.

## Results

### General characteristics of the study population

The socio-demographic characteristics of the students surveyed are summarized in Table 1. A total of 263 students were interviewed, of which 72 (27.4%) were female and 191 (72.6%) were male. The median age was 23 years old. The minimum age of respondents was 16 years and the maximum was 45 years. Respondents living in on-campus Aborney-Calavi housing represented (69.6%; n=183), followed by those living in on-campus Cotonou housing (26.2%; n=70). Regarding the field of study, 12.2% (n=32) of the respondents were studying human health compared to 9.5% (n=25) and 78.3% (n=206), respectively, enrolled in animal health and other science fields (social sciences, economics and management sciences, legal sciences, industrial sciences other than health, chemical and pharmaceutical sciences, business, etc.). Only 56 (21.3%) students reported to be a primary owner of at least one dog. In the dog owning group, the median number of dogs per household was 1 with a maximum of 6 and a minimum of 1. Also in this group, local breed dogs were the most frequently mentioned (62.5%; n=35) or followed by mixed-breed dogs (23.2%; n=13). These dogs lived with the students as companion animals (33.9%; n=19) or for security reasons (41.1%; n=23). Only 33 out of 56 (58.9%) of student dog owners had a kennel. However, 45 (80%) of the 56 dog owners said that their dogs were always locked up at home and 21 (37.5%) said that they were the only person who looked after their dog(s). Also, in this group of 56 student dog owners, 26 (46.4%) reported that their dogs were aggressive by nature.

**Table 1:** socio-demographic characteristics of students surveyed at the Abomey-Calavi and FSS Cotonou campuses and their level of knowledge attitudes and practices on rabies in 2020

Variables	Number and percentage (%) of respondents	Knowledge No. of respondents (%)		Attitudes and Practices No. of respondents (%)	
		Inadequate	Adequate	Inadequate	Adequate
**Sex**					
Female	72 (27.4)	25 (34.7)	47 (65.3)	26 (36.1)	46 (63.9)
Male	191 (72.6)	69 (36.1)	122 (63.9)	73 (38.2)	118 (61.8)
**Field of study**					
Students in human health	32 (12.2)	4 (12.5)	28 (87.5)	5 (15.6)	27 (84.4)
Students in animal health	25 (9.5)	3 (12)	22 (88)	5 (20)	20 (80)
Students in other disciplines	206 (78.3)	87 (42.2)	119 (57.8)	89 (43.2)	117 (56.8)
**Age**					
≤ 23 years old	197 (74.9)	49 (32.2)	103 (67.8)	57 (37.5)	95 (62.5)
>23 years old	66 (25.1)	45 (40.5)	66 (59.5)	42 (37.8)	69 (62.2)
**Location of Primary Residence**					
Calavi	184 (69.9)	64 (34.8)	120 (65.2)	74 (40.2)	110 (59.8)
Cotonou	69 (26.2)	28 (40.6)	41 (59.4)	23 (33.3)	46 (66.7)
Autres	10 (3.9)	2 (20)	8 (80)	2 (20)	8 (80)
**Number of persons in household**					
1-3	89 (33.8)	26 (29.2)	63 (70.8)	31 (34.8)	58 (65.2)
4-6	139 (52.8)	56 (40.3)	83 (59.7)	53 (38.1)	86 (61.9)
≥7	35 (13.4)	12 (34.3)	23 (65.7)	15 (44.1)	19 (55.9)
**Owner of dog(s)**					
Yes	56 (21.3)	15 (26.8)	41 (73.2)	25 (44.6)	31 (55.4)
No	207 (78.7)	79 (38.2)	128 (61.8)	74 (35.7)	133 (64.3)

### Knowledge: general description, and correlates of the level of knowledge

Table 2 and Table 2 (suite) show the knowledge of students surveyed about rabies. In total, 53.2% (n=140) of students have heard of dog rabies while 46.8% (n=123) have never heard of it. Regarding human rabies, 47.5% (n=125) have heard of it while 52.5% (n=138) had not. Regarding the causes of canine rabies, 45.2% (n=119) mentioned the rabies virus, 28.9% (n=76) considered it as a mental illness, malnutrition or poisoning. In relation to the causes of human rabies, 53.2% (n=140) mentioned that the disease is transmissible from dog to man, and 18.6% (n=49) by a mental illness. The results revealed that 49.0% (n=129) of the respondents recognized stray dogs as the main source of rabies infection and 4.6% (n=12) noted bats. Investigations into the modes of transmission showed that, dog bites were cited as the main source by 41.4% (n=109) of the respondents, whereas scratches by dogs were cited by 2.7% (n=7) and salivary transmission through licking by 0.4% (n=1). Only 23.6% (n=62) of the students said that the dog was a reservoir of rabies infection and 7.6% (n=20) said that the bat was a reservoir of the rabies virus. Regarding the age groups of people most exposed to this disease, the study revealed that 62% (n=163) of the students reported that everyone was exposed to the disease equally, and 30.8% (n=81) for the age group (0-15 years). Analyses showed a lack of knowledge among students about the consequences of animal and human rabies. Indeed, concerning the perceived consequences of human rabies, 37.6% (n=99) said that death was the outcome, while 3.4% (n=9) reported healing and 1.9% (n=5) reported paralysis. Compared to the consequences of canine rabies on the dog, 26.2% (n=69) declared death as an outcome, 7.2% (n=19) recovery and 2.3% (n=6) paralysis. The survey also revealed that the students had ideas of how best to combat this disease. Among them, 32.7% (n=86) had mentioned public awareness and education about dog vaccines, 20.5% (n=54) for simultaneous euthanization of stray dogs as well as the promotion of public awareness and education about dog vaccines, 20.2% (n=53) for the vaccination of dogs only, and 10.3% (n=27) for public education only. Table 3 shows the relationship between socio-demographic characteristics and the knowledge of students on the Abomey-Calavi and Cotonou campuses. The study concluded that age, field of study and dog ownership influenced the students´ knowledge of rabies. Students in human or animal health had 3 times more knowledge on rabies (OR<1; p<0.05).

**Table 2:** knowledge of students surveyed about rabies at the Abomey-Calavi and Faculty of Health Sciences Cotonou campuses in 2020

Variables	Number of respondents	Percentage (%)
**Heard about canine rabies**		
Yes	140	53.2
No	123	46.8
**Heard about human rabies**		
Yes	125	47.5
No	138	52.5
**Cause of canine rabies**		
Rabies virus	119	45.2
Mental illness or malnutrition or poisoning	76	28.9
Don’t know	68	25.9
**Cause of human rabies**		
Disease transmitted from dogs to humans	140	53.2
Mental illness only	49	18.6
Poisoning or malnutrition	6	2.3
Don´t know	68	25.9
**The perceived most likely source of the rabies virus**		
Stray dogs	129	49.0
Owned dogs	10	3.8
Cats	2	0.8
Bats	12	4.6
Cat, Bat, and Stray dog	10	3.8
Bat and stray dog	17	6.5
Don´t know	83	31.6
**Suspected modes of viral transmission**		
Bites	109	41.4
Scratches	7	2.7
Licks	1	0.4
Licks and Bites	6	2.3
Scratches and Bites	32	12.2
Scratches, Licks, and Bites	43	16.3
Consumption of dog meat	12	4.6
Don´t know	53	20.2
**Reservoirs**		
Dogs	62	23.6
Cats	3	1.1
Bats	20	7.6
Snakes	6	2.3
Cats, Bats and Dogs	20	7.6
Others	152	57.8
**Most exposed age groups**		
0-15 years	81	30.8
16-25 years	9	3.4
26-35 years	3	1.1
≥35 years	7	2.7
Everybody	163	62.0
**Consequence of human rabies**		
Death	69	26.2
Healing	19	7.2
Paralysis	6	2.3
Don´t Know	169	64.3
**Perceived consequence of canine rabies**		
Death	99	37.6
Healing	9	3.4
Paralysis	5	1.9
Don´t Know	150	57.0
**Perceived best means of rabies control**		
Promotion of educating dog owners about dog vaccines and public awareness about rabies	86	32.7
Euthanization of stray dogs and simulateneous public education campaigns about dog vaccines	54	20.5
Dog vaccination only	53	20.2
Public education about rabies only	27	10.3
Don´t know	43	16.3

**Table 3:** attitudes and practices of students surveyed about rabies at the Abomey-Calavi and Faculty of Health Sciences Cotonou campuses in 2020

Variables	Number of respondents		Percentage (%)
**A person´s first action after dog bite**	****		****
Go to the human hospital only	115		43.7
Go to the veterinary hospital only	17		6.5
Go to the hospital then consult a veterinarian for the dog	30		11.4
Clean the wound with soap and water only	12		4.6
Clean wound then go to the hospital then consult a veterinarian for the dog	26		9.9
Clean wound then go to the hospital	27		10.3
Use medicinal plants with traditional healer	10		3.8
Do nothing	12		4.6
Other	14		5.3
**Best first destination for a person after been bitten by a dog**			
Human hospital	159		60.5
Traditional healer	11		4.2
Veterinary hospital	12		4.6
Traditional healer and human hospital	5		1.9
Other	76		28.9
**Expected treatment for rabies by traditional healer**			
Cola	17		6.5
Black stone	10		3.8
Cassava leaf	9		3.4
Don´t know	227		86.3
**Attitudes towards a biting dog**			
Take the dog to a veterinarian	72		27.4
Euthanize the dog	49		18.6
Do nothing	72		27.4
Chase the dog away from the house	16		6.1
Don´t know	54		20.5
**Perceived consequences of inaction after a dog bite**			
Person gets rabies	153		58.2
Person gets tetanus	5		1.9
Person gets rabies and tetanus	31		11.8
No consequences expected	10	3.8	
Don´t know	64	24.3	
**Reasons for stopping rabies post-exposure treatment**			
Lack of finances	184	70.0	
Without reason	21	8.0	
Don´t know	58	22.1	

### Attitude and practices: general description, then correlates

**Figure 1:**
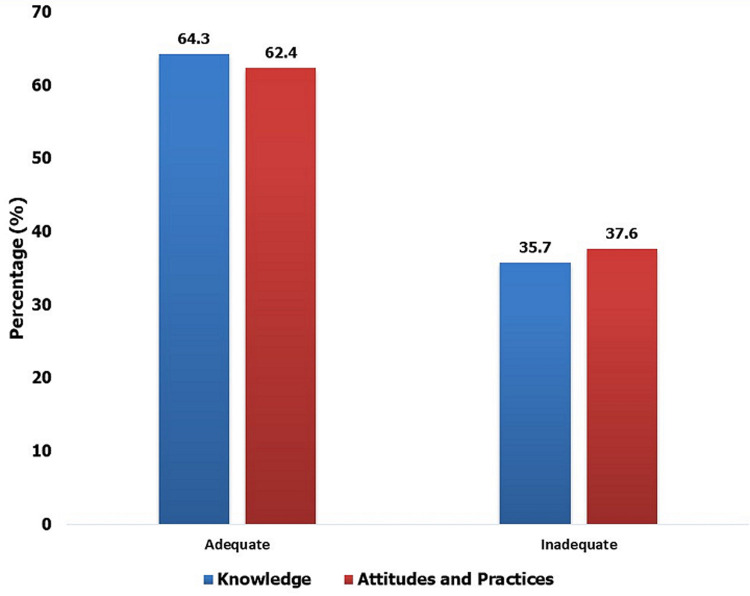
results of the overall assessment of students' knowledge, attitudes and practices in the study area

Table 4 shows the attitudes and practices adopted by students in the event of a dog bite. In addition, this table shows 43.7% (n=115) of respondents felt that they should first go to a health centre in case of a dog bite. Concerning the choice between first stopping at an animal hospital or human hospital, 60.5% (n=159) of respondents would first visit a human clinic and 4.6% (n=12) would directly go to a veterinary hospital after getting a dog bite. Specifically, 100% (n=263) of human medical students said they would first go to a human hospital whereas some veterinary students said that they would first go to a veterinary clinic. Meanwhile, 1.9% (n=5) of all respondents opted to go to both traditional healers and western medicine hospitals and 28.9% (n=76) chose other places. Of the expected recipes used by traditional healers, 6.5% (n=17) mentioned cola, 3.8% (n=10) mentioned black stone, and 3.4% (n=9) mentioned cassava leaves. Regarding the fate of the biting dogs, 27.4% (n=72) said they would take the dog to a veterinarian. Regarding the perceived consequences of inaction after a dog bite, 58.2% (n=153) mentioned the risk of developing rabies. Among the students interviewed on the reasons for stopping rabies post-exposure treatment for themselves, 70.0% (n=184) mentioned a lack of financial means. Table 5 shows relationship between socio-demographic characteristics and the attitudes and practices of students on the Abomey-Calavi and Cotonou campuses. This shows that the field of study influenced the practices and attitudes of students on the Abomey-Calavi and Cotonou campuses with respect to rabies. Students in human or animal health were 3 times more aware on rabies compared to others (OR<1; p<0.05). Figure 1 presents the overall assessment of students´ knowledge, attitudes and practices about rabies at the Abomey-Calavi campus and the Faculty of Health Sciences in Cotonou. The results of the assessment of students´ knowledge, attitudes and practices regarding rabies. The analysis revealed that the surveyed students from the Abomey-Calavi campus and the Cotonou Faculty of Health Sciences had good knowledge (64.3%; n=169) and attitudes and practices (63.4%; (n=167)) on rabies.

**Table 4:** relationship between socio-demographic characteristics and the students’ knowledge of rabies at the Abomey-Calavi and Cotonou campuses

Variables	Univariable Analysis		Multivariable Analysis	
**OR (95% CI)**	**P-value**	**OR (95% CI)**	**P-value**
Sex	0.9407 (0.5331-1.6600)	0.833		
Field of study	0.3720 (0.2164-0.6395)	0.0003	0.3543 (0.2058-0.6097)	0.0002
Age	1.4332 (0.8613-2.3848)	0.1659	1.7314 (1.0095-2.9695)	0.0461
Location of Primary Residence	0.9896 (0.6247-1.5675)	0.9643		
Number of persons in the household	0.8378 (0.5685-1.2348)	0.3712		
Dog owners	1.6867 (0.8765-3.2455)	0.1175	1.9207 (0.9627-3.8318)	0.064

**Table 5:** relationship between socio-demographic characteristics and the students´ attitudes and practices about rabies at the Abomey-Calavi and Cotonou campuses

	Univariable Analysis		Multivariable Analysis	
	OR (95% CI)	P-value	OR (95% CI)	P-value
Sex	0.9141 (0.5208-1.6042)	0.7543		
Field of study	0.4557 (0.2839-0.7316)	0.0011	0.4560 (0.2825-0.7359)	0.0013
Age	1.0145 (0.6123-1.6809)	0.9554		
Location of Primary Residence	1.4440 (0.8939-2.3325)	0.1332	1.4146 (0.8708-2.2981)	0.1612
Number of persons in the household	0.8333 (0.5681-1.2223)	0.3508		
Dog owners	0.6899 (0.3792-1.2554)	0.2243		

## Discussion

In our report focusing on knowledge, attitudes, and practices of students in Benin on rabies, males were interviewed more often than females since they were encountered more frequently during the survey period. The participants´ median age was of 23. By this age, the authors would have expected the respondents to be sufficiently aware of the risks associated with dog bites, and that this knowledge would limit their exposure to rabies. However, this was not found to be the case. Regarding information on rabies, around half of the respondents stated that they had heard of rabies. These results are quite different to those obtained by Adjé *et al*. [9] in Senegal who showed that 96.5% of students surveyed at University Cheikh Anta Diop de Dakar - Sénégal (UCAD) had heard of rabies at least once. Furthermore, the proportion of knowledgeable respondents in Benin is lower than another study performed in Grenada where 97.6% of households reported that they had heard of animal rabies [10]. This result may be due to the lack of a rabies awareness and education campaigns among students in Benin. It will therefore be necessary to intensify awareness and education campaigns among students who could be a good relay of information to other members in their communities as seen in Borries *et al*. [11]. Furthermore, the rabies virus was recognized by 45.2% of respondents in Benin as the cause of canine rabies and 53.2% also recognized that human rabies is a disease transmitted from dog to human. This rate is higher than the 41.43% respondent rate found in a recent study conducted in Chad by Mindekem *et al*. [3].

In this study, the dog bite was recognized as the main mode of rabies transmission. The percentage of respondents who recognized the infected dog as the main source of rabies transmission is higher than that obtained in study of Khan *et al*. [12] where 2.9% of participants were aware that a bite from a rabid dog was the main source of rabies. In addition, a study conducted in Bangladesh by Mujibur Rahaman *et al*. [13], showed that 58.97% of the respondents recognized a dog bite as the predominant mode of rabies transmission. Scratching and licking were less recognized as modes of viral transmission in this study. This result is seemingly due to their lack of knowledge in the health field since 100% of medical and majority of veterinary students understood rabies risks. Regarding rabies knowledge about the most-at-risk age groups, most participants thought that all age groups were equally at risk; this observation was also made by Mindekem *et al*. [3]. While it is well-established that people under the age of 15 are the most at-risk with regard to acquiring rabies [2,5], our study identified only 30.8% of respondents knew this fact. This finding is lower than previously reported elsewhere [3,5]. This low level of knowledge reflects the ignorance in the general population and highlights that lack of childhood and public education is a substantial public health concern. However, despite this ignorance, death was recognized as an outcome by the students for the selected consequence of human rabies (26.2%) as well as for canine rabies (37.6%). This small fraction of respondents was well-aware of the danger of this disease. Compared to the study of Ahmed *et al*. [14] in India, where 72.4% of respondents were aware that rabies is a fatal disease, the proportion of recognition in Benin is markedly less.

In relation to the means of rabies control, most respondents stated that it was necessary to raise public awareness about dog vaccinations in order to decrease the occurrence of rabies transmission. These suggestions are consistent with those made by Chadian respondents [3]. In summary, our current study shows that 64.3% of respondents have a sufficient knowledge of rabies. This proportion is higher than findings obtained in Senegal (22.4%) which was evaluated by Hagos *et al*. [15] and Niang *et al*. [16], but is lower than findings obtained in Tanzania by Sambo *et al*. [17]. Concerning actions to take after a dog bite, the vast majority of students were unaware that the bite wound should be immediately and thoroughly cleaned with soap and water as a first line of defense against infection. This ignorance is of significant public health concern. The percentage of respondents who were unaware of the best first course of action after being bitten by a dog in our study is higher compared to other countries such as Bangladesh (2%), Cameroon (18%), and Ethiopia (30.7%) [18].

Finances affect decision making. For some respondents, it is preferable to go to traditional healers as western medicine hospitals cost more. Similarly, the majority of respondents reported that they would likely stop their rabies post-exposure treatment (PEP) due to a lack of finances. This finding is not unique to Benin. Other studies conducted in India and Senegal had respondents report poor compliance with PEP treatment due to the cost [19,20]. Due to this financial burden, the majority of our study´s respondents noted that using traditional recipes which lack empirical evidence of successful rabies treatment increases the risk of a community´s rabies incident. Regarding the fate of the biting dog, 27.4% said they would take it to a veterinarian. This result is lower compared to that obtained by Tiembré *et al*. [21] in Côte d'Ivoire where 64.5% of people had decided to catch the dog and send it to the veterinarian for observation. In conclusion, the authors are in agreement with the findings of Borries *et al*. [11] and Thomson [22] in that the control of this diseases such as rabies requires heightened public awareness and education in order to change behavior and reduce risk of disease transmission. Our study has several limitations. The number of participants was quite small and respondents cannot be considered as representative of the whole population. However, it has identified the gaps in students´ knowledge, attitudes and practices about effective rabies prevention and control.

## Conclusion

Rabies remains a little-known disease among University of Abomey-Calavi students. Of the students surveyed, around half have heard of both canine and human rabies. In addition, the stray dog was recognized as the main source of infection for this disease through bites by the respondents. In the event of a dog bite, only a minority of students knew that the wound should be cleaned immediately. Finally, public awareness through vaccination and education campaigns was recognized as the most efficient measure to control rabies. In sum, this study has identified increased risks of people getting rabies through evaluation of answers to the questions pertaining to the respondent´s attitude and practices. In light of these findings, the implementation of a strategic plan for effective rabies control is necessary for its eradication in Benin. This control requires intensifying public awareness and public rabies education, especially with students.

### What is known about this topic

- Rabies is a vaccine-preventable viral zoonotic disease. Once clinical symptoms appear, rabies is fatal in almost 100% of cases;
- In Africa, and especially in Benin, rabies cases are dog-mediated and the burden of disease is disproportionally seen in rural and impoverished communities.

### What this study adds

- This study identifies the gaps in public knowledge about effective rabies prevention and control;
- It found that people who seek and receive treatment with traditional care takers have an increased risk of acquiring rabies after a dog bite;
- Majority of respondents claimed they would visit a western medicine human clinic immediately after a dog bite. They recognized that inaction after a dog bite may increase the risk of a person getting rabies.
